# Phosphatidic acid, a versatile water-stress signal in roots

**DOI:** 10.3389/fpls.2013.00525

**Published:** 2013-12-23

**Authors:** Fionn McLoughlin, Christa Testerink

**Affiliations:** Section of Plant Physiology, Swammerdam Institute for Life Sciences, University of AmsterdamAmsterdam, Netherlands

**Keywords:** phosphatidic acid, *Arabidopsis thaliana*, roots, salt, drought, protein kinase

## Abstract

Adequate water supply is of utmost importance for growth and reproduction of plants. In order to cope with water deprivation, plants have to adapt their development and metabolism to ensure survival. To maximize water use efficiency, plants use a large array of signaling mediators such as hormones, protein kinases, and phosphatases, Ca^2^^+^, reactive oxygen species, and low abundant phospholipids that together form complex signaling cascades. Phosphatidic acid (PA) is a signaling lipid that rapidly accumulates in response to a wide array of abiotic stress stimuli. PA formation provides the cell with spatial and transient information about the external environment by acting as a protein-docking site in cellular membranes. PA reportedly binds to a number of proteins that play a role during water limiting conditions, such as drought and salinity and has been shown to play an important role in maintaining root system architecture. Members of two osmotic stress-activated protein kinase families, sucrose non-fermenting 1-related protein kinase 2 and mitogen activated protein kinases were recently shown bind PA and are also involved in the maintenance of root system architecture and salinity stress tolerance. In addition, PA regulates several proteins involved in abscisic acid-signaling. PA-dependent recruitment of glyceraldehyde-3-phosphate dehydrogenase under water limiting conditions indicates a role in regulating metabolic processes. Finally, a recent study also shows the PA recruits the clathrin heavy chain and a potassium channel subunit, hinting toward additional roles in cellular trafficking and potassium homeostasis. Taken together, the rapidly increasing number of proteins reported to interact with PA implies a broad role for this versatile signaling phospholipid in mediating salt and water stress responses.

## INTRODUCTION

Plants have to adapt to various changes in their environment and signals from the outside have to pass the membrane in order for the cell to respond. Environmental stress causes changes in the phospholipid composition of cellular membranes. Several lipids, which are only present in small amounts under normal conditions, are synthesized rapidly and transiently in response to stress. They act as a lipid second messenger and can form docking sites that bind different proteins and thus provide spatial and transient signals needed to adequately respond to external stimuli (Meijer and Munnik, 2003; Wang, 2004; Munnik and Testerink, 2009; Xue et al., 2009; Munnik and Vermeer, 2010). The phospholipid phosphatidic acid (PA) is one of these signaling lipids, which accumulates rapidly in response to different environmental signals (Testerink and Munnik, 2005; Li et al., 2009; Galvan-Ampudia and Testerink, 2011).

## THE INVOLVEMENT AND GENERATION OF PA DURING ABIOTIC STRESS AND DEVELOPEMENT

Induced PA formation has been described as a response of plants to abiotic stress stimuli such as dehydration (Jacob et al., 1999), salt and osmotic stress (Munnik et al., 2000) treatment with the plant hormone abscisic acid (ABA; Fan et al., 1997; Ritchie and Gilroy, 1998; Jacob et al., 1999) and also accumulates in response to biotic stress stimuli (van der Luit et al., 2000; de Torres Zabela et al., 2002; de Jong et al., 2004).

### LIPIDOLOGY AND PA-GENERATING ENZYMES

Different PA metabolizing pathways have been shown to contribute to PA production in response to abiotic stress (Munnik et al., 2000; Ruelland et al., 2002; Arisz et al., 2009; Bargmann et al., 2009; Li et al., 2009; Hong et al., 2010). The phospholipase D (PLD) enzyme hydrolyses primarily structural lipids such as phosphatidylcholine (PC), and phosphatidylethanolamine (PE), resulting in formation of PA and the remaining headgroup (Pappan et al., 1998). Phospholipase C (PLC) hydrolyses phosphatidylinositol lipids (PPIs) into water soluble inositol-bis or trisphosphate (IP_2_, IP_3_) and diacylglycerol (DAG), which remains in the membrane (Munnik and Vermeer, 2010). DAG can be subsequently phosphorylated to PA by DAG kinase (DGK; Arisz et al., 2009).

Twelve PLDs have been identified in the model plant species *Arabidopsis*, which were initially classified in two groups based on their N-terminal lipid-binding domain. These domains consist either of a pleckstrin homology (PH) and PHOX (PX) or a calcium dependent-lipid binding (C2) domain (Elias et al., 2002). Later, these classes were further subdivided into six classes based on sequence homology and *in vitro* enzymatic activity: three α-, two β-, three γ-, one δ-, and one ε- PLD with a C2 domain and two ζ-class PLDs that contain PH and PX domains (Qin and Wang, 2002; Bargmann and Munnik, 2006; Li et al., 2009).

In the *Arabidopsis* genome, nine PLCs and seven DGK genes have been identified. Initially, PLC/DGK derived PA was primarily implicated in responses to biotic stress (van der Luit et al., 2000; de Jong et al., 2004). However, abiotic stress, in particular cold stress, also induced an accumulation of PLC/DGK-mediated PA formation (Ruelland et al., 2002; Gomez-Merino et al., 2004). The DGKs involved in PA formation during cold stress have not been identified yet. T-DNA insertion lines of the seven DGKs did not alter PA formation in response to cold, which is likely due to redundancy (Arisz et al., 2013) and therefore the role of DGK-derived PA in response to abiotic stress remains largely unknown.

### GENETIC EVIDENCE FOR PLD REQUIREMENT DURING WATER STRESS AND RELATED RESPONSES OF ROOTS

**FIGURE 1:**
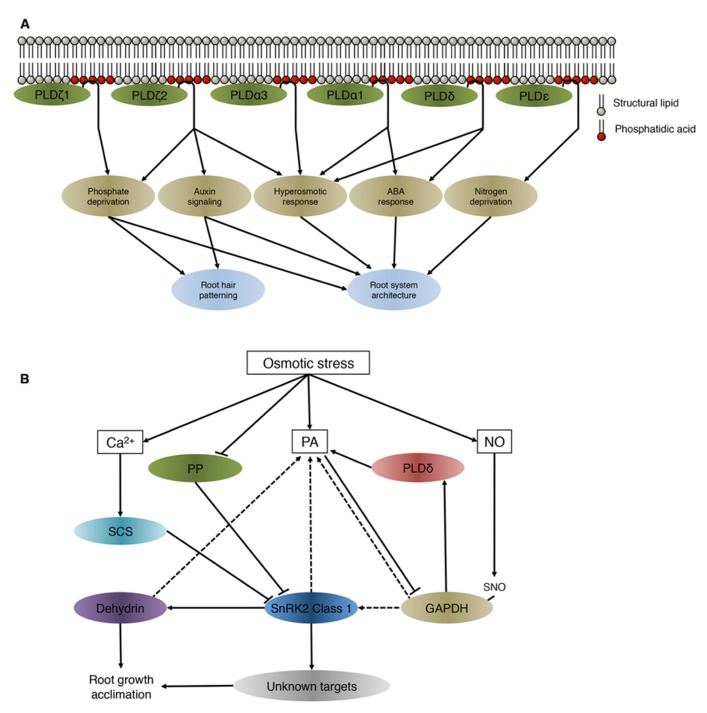
**(A)** PA derived from different PLDs involved in the maintenance of root system architecture during abiotic stress. PLDs regulate downstream targets through producing PA. Although all PLD isoforms hydrolyse structural lipids and generate PA *in vitro*, they have been identified to be involved in different processes and signaling cascades *in vivo*. **(B)** Preliminary network of osmotic stress-induced PA-SnRK2 signaling cascades in roots. This model is based on data obtained on the class 1 SnRK2 members in different plant species. SnRK2, sucrose non-fermenting 1-related protein kinase 2; SCS, SnRK2-interacting calcium sensor; PA, phosphatidic acid; NO, nitric oxide; SNO, *S*-nitrosylated; PP, protein phosphatase; GAPDH, glyceraldehyde-3-phosphate; PLDδ, phospholipase D δ. Solid lines indicate an activation or inhibitory effect, dashed lines show which proteins/lipids interact without a direct change in activity.

Roots are the primary site of perception of salt stress, drought and low nutrient availability. To cope with these conditions, plants adapt the growth and morphology of their roots. Several PLD isoforms were found to be involved in adjusting root system architecture during abiotic stress (Galvan-Ampudia and Testerink, 2011; **Figure 1A**).

PLDζ2 is involved in directional root growth in saline conditions. Exposing one side of the root to salt increased pin-formed (PIN) 2 internalization, effectively redistributing auxin in the root tip. This redistribution resulted in bending away from saline conditions, named halotropism. A *pldζ2*-KO (knock-out) mutant** showed reduced clathrin-dependent PIN2 internalization and reduced primary root bending (Galvan-Ampudia et al., 2013). Expression of PLDζ2 increased under low phosphate availability (Oropeza-Aburto et al., 2011) and the *pldζ2*-KO showed increased root hair growth when deprived of phosphate (Cruz-Ramirez et al., 2006). In accordance, less PA was formed in low phosphate conditions in the *pldζ1/2* double mutant (Li et al., 2006a) and this mutant showed reduced lateral root and increased primary root growth in low phosphate conditions (Li et al., 2006b). In addition, the *pldζ2*-KO mutant also exhibited decreased sensitivity to auxin and a reduced root gravitropic response (Li and Xue, 2007).

Nitrogen is another important nutrient for plants and *Arabidopsis* PLDε-OE lines displayed an increase in lateral root and root hair elongation and primary root growth in low nitrogen conditions. This effectively increased the dry weight of the plant under these conditions and indicated an important role for PLDε in growth and nitrogen signaling (Hong et al., 2009).

PLDα1 and PLDδ are involved in different responses to abiotic stress including reactive oxygen species (ROS) signaling in response to ABA (Sang et al., 2001; Zhang et al., 2003; Zhang et al., 2009; Uraji et al., 2012) studied in stomata and leaves. The same phospholipases were also shown to play distinct roles in freezing tolerance (Welti et al., 2002; Li et al., 2004). Expression of PLDδ was elevated in response to dehydration and high salt stress (Katagiri et al., 2001). Salt stress induced formation of PA through PLDα1 and PLDδ, where both single mutants showed a reduction in primary root growth in saline conditions and during dehydration. This was even clearer in the *pldα1/δ* double mutant (Bargmann et al., 2009). A similar reduction in root growth was observed in *pldα3*, which was more susceptible to salinity and water deficiency. In hyperosmotic-stress conditions, the *pldα3* mutant displays a reduction in primary root growth and a reduction in lateral roots (Hong et al., 2008).

Together, these studies show that PLDs are important for maintaining root growth in saline and hyperosmotic stress conditions amongst other functions. PA is not limited to its function as a signaling lipid; it is also an important intermediate during lipid-turnover. Therefore it is hard to separate the role of PA in lipid-turnover from its role in signaling and protein recruitment (Testerink and Munnik, 2011). To discern between the different roles of PA it is important to identify which proteins interact with PA and how these mediate the response that eventually leads to the acclimation to different stresses (Testerink and Munnik, 2005). In contrast to other signaling lipids such as phosphoinositides, no consensus PA-binding domain has been identified, which hampers the identification of new PA targets. A number of PA binding proteins have been identified involved in diverse cellular processes (**Table 1**).

**Table 1:** An overview of PA targets identified in plants.

PA targets	Function	Role in root growth?	Reference
PDK1	Root hair development, defense to pathogens	Yes	Deak etal. (1999), Anthony etal. (2004, 2006)
ABI1	ABA signaling	Yes	Zhang etal. (2004)
Dehydrins	Protection during abiotic stress	Yes	Koag etal. (2003, 2009), Eriksson etal. (2011)
SnRK2.10/2.4	Salt stress signaling	Yes	Testerink etal. (2004), McLoughlin etal. (2012)
RCN1	Auxin transport, ethylene signaling	Yes	Testerink etal. (2004), Gao etal. (2013)
PID	PIN localization	Yes	Zegzouti etal. (2006)
CP	Actin polymerization	Yes	Huang etal. (2006), Li etal. (2012), Pleskot etal. (2012)
TGD2	Lipid transport	Not reported	Awai etal. (2006)
AGD7	ER/Golgi trafficking	Not reported	Min etal. (2007)
CTR1*	Ethylene signaling	Yes	Testerink etal. (2007)
TaPEAMT1/2	Lipid metabolism	Not reported	Jost etal. (2009)
RbohD/F*	Oxidative stress	Yes	Zhang etal. (2009)
PEPC	Metabolism	Not reported	Testerink etal. (2004), Monreal etal. (2010)
MPK6	Abiotic and biotic stress signaling	Yes	Yu etal. (2010)
MGD1	Lipid metabolism	Not reported	Dubots etal. (2010)
ZmCPK11	Protein kinase	Not reported	Klimecka etal. (2011)
SPHK1	Sphingosine kinase	Yes	Guo etal. (2011)
TGD4	Lipid transport	Not reported	Wang etal. (2012, 2013)
PTEN2A	Lipid phosphatase activity	Not reported	Pribat etal. (2012)
CdeT11-24	Protein protection	Not reported	Petersen etal. (2012)
14-3-3 protein	Protein binding	Not reported	Camoni etal. (2012)
MAP65-1	Microtubule organization	Yes	Zhang etal. (2012)
GAPC	Metabolism	Yes	McLoughlin etal. (2013), Kim etal. (2013)

Abbreviations of all the PA targets identified in different parts of the plant are given (left column) with their putative function (2nd column), whether there is a known effect on root growth (3rd column) and references for the PA-binding evidence (right column). PDK1, phosphoinositide-dependent kinase 1; ABI1, ABA insensitive 1; SnRK2.10, sucrose non-fermenting 1-related protein Kinase 2.10/2.4; RCN1, roots curl in NPA 1; PIN1, PIN-formed 1; PID, PINOID; CP, heterodimeric actin capping protein; tGD2/4, trigalactosyldiacylglycerol 2/4; AGD7, Arf gap domain 7; CTR1, constitutive triple response 1; TaPEAMT1/2, phosphoethanolamine *N*-methyltransferase; RbohD/F, respiratory burst oxidase homolog D/F; PEPC, phosphoenolpyruvate carboxylase; MPK6, MAP kinase 6; MGD1, monogalactosyl diacylglycerol synthase 1; ZmCPK11, calcium-dependent protein kinase11; SPHK1, SPHingosine kinase1; PTEN, phosphatase and TENsin homolog deleted on chromosome 3; MAP 65-1, microtubule-associated proteins 65-1; GAPC, glyceraldehyde-3-phosphate dehydrogenase C. *Evidence for a role in root growth: CTR1 (Kieber etal., 1993), RbohD/F (Ma etal., 2012).

## PA PROTEIN TARGETS INVOLVED IN OSMOTIC/SALT STRESS SIGNALING AND ROOT SYSTEM ARCHITECTURE

### METABOLISM

A central mediator in metabolism, glyceraldehyde-3-phosphate dehydrogenase (GAPDH), is targeted to PA in response to salt in *Arabidopsis* roots (McLoughlin et al., 2013). The best-described role of GAPDH is the conversion of glyceraldehyde-3-phosphate to D-glycerate 1,3-bisphosphate in the glycolytic breakdown of glucose. PA-binding does not alter the activity of GAPDH dramatically *in vitro* but adding PA to seedlings did induce proteolytic cleavage of glyceraldehyde-3-phosphate dehydrogenase C2 (GAPC2; Kim et al., 2013). Adding exogenous PA also reduced primary root growth, which was more severe when GAPDH was over-expressed whilst knock-out mutants showed less reduction in growth (Kim et al., 2013) indicating that the effect of PA on root growth was partially mediated by proteolytic degradation of GAPDH. Although GAPDH has been described to be involved in different non-metabolic processes, promoting its degradation might mediate energy conservation and arrest of root growth, which are immediate and relevant responses to any osmotic stress including saline conditions (Munns and Tester, 2008).

### CYTOSKELETON AND MEMBRANE CELLULAR TRAFFICKING

Phosphatidic acid has also emerged as an important regulator of microtubules and actin organization and re-organization. Microtubule reorganization is crucial for plants to adapt to saline conditions (Wang et al., 2007). In the *pldα1* mutant background, the microtubule disorganization was more severe in response to salt and could not be recovered after the removal of salt. This effectively resulted in the plant being more salt sensitive in a microtubule-associated proteins 65-1 (MAP65-1) dependent manner. PA directly interacted with MAP65-1 and promoted the interaction of MAP65-1 with microtubules, effectively promoting the polymerization of cortical microtubules (Zhang et al., 2012).

In addition to microtubules, PA levels were also shown to be important for the behavior of actin filaments through the regulation of actin capping proteins (Li et al., 2012). PA specifically interacts with an actin capping protein (CP; Huang et al., 2006), which prevents actin filament from annealing and elongating. PA-binding reduced the activity of CP, effectively promoting actin reorganization and promoting cytoskeleton dynamics which are required for adaptation to adverse conditions.

In mammalian cells, PA and PA-generating enzymes such as PLD and DGK have been implicated in various aspects of vesicle transport (Manifava et al., 2001; Corda et al., 2002; Jang et al., 2012), but so far, little evidence is present that suggest a similar role in plants. Recently, clathrin heavy chain and clathrin assembly units were shown to recruit to the membrane in *Arabidopsis* roots in response to salt and to bind to PA-beads (McLoughlin et al., 2013). This likely represents an important aspect of the molecular mechanism of salt-induced PIN2 internalization which controls directional root bending in saline conditions (Galvan-Ampudia et al., 2013).

### CELLULAR SIGNALING AND DEVELOPMENT

The *Arabidopsis* phosphoinositide-dependent kinase 1 (PDK1) binds several phosphoinositides and PA through its PH domain (Deak et al., 1999). PA activates PDK1 and indirectly, its downstream target AGC2-1 (OXI1) (Anthony et al., 2004, 2006). This signaling cascade induces a respiratory burst required for the full activation of mitogen activated protein kinase 6 (MPK6; Rentel et al., 2004), which was also shown to bind PA (Yu et al., 2010). MPK6 knock-out plants showed reduced growth in the primary root in saline conditions and the activation of MPK6 in response to salt is abolished in the *pldα1* mutant background. In addition, MPK6 can phosphorylate the Na^+^/H^+^ antiporter salt overly sensitive (SOS1) *in vitro* and might therefore play a direct role in sodium homeostasis in roots (Yu et al., 2010). Another phosphorylation target of PDK1, PINOID (PID), was shown to bind phosphoinositides and PA. PID is involved in the asymmetric distribution of PIN auxin transporters, which are key regulators of root development (Zegzouti et al., 2006). Additionally, PA was also shown to bind the subunit of protein phosphatase 2A (PP2A); roots curl in NPA 1 (RCN1; Testerink et al., 2004). Recruitment to the membrane increased the activity of PP2A (Gao et al., 2013). PP2A in turn dephosphorylates the auxin transporter PIN1, changing its polarity and effectively altering the auxin distribution in the root, which is necessary for normal root development (Gao et al., 2013).

In a proteomics screen to identify PA targets during salt stress in *Arabidopsis* roots, the potassium channel *β* subunit KAB1 was shown to recruit to the membrane in response to salt and bind to PA (McLoughlin et al., 2013). The *Arabidopsis* genome encodes a single potassium channel *β* subunit 1 (Tang et al., 1995), which, as a tetramer, associates with the transmembrane *α* subunits of KAT1 channels (Tang et al., 1996). KAT1 is inactivated rapidly and internalized in response to ABA in guard cells. The inactivation occurs more rapidly than internalization, which suggests an additional mechanism. PA-recruitment could play a role in the inactivation of KAT1 by competing for KAB1, but this remains to be investigated. Since AtKAT1 expression is prevalent in leaves rather than roots, KAB1 is speculated to bind not exclusively to KAT1 but also to a different *α* subunit, *viz.* AKT1, which is selectively expressed in *Arabidopsis* roots (Tang et al., 1996). Recruitment of KAB1 by PA opens the possibility that PA might function as a regulator of potassium homeostasis in different parts of the plant.

The sucrose non-fermenting 1-related protein kinase 2 proteins (SnRK2s), is a family of osmotic stress-activated protein kinases (Kulik et al., 2011). SnRK2.10, a member of the class 1 subfamily, which is activated by salt and water stress but not by ABA, was identified in a PA affinity screen (Testerink et al., 2004). More recently, both SnRK2.10 and its close homolog SnRK2.4 were shown to bind PA directly. *Arabidopsis* *snrk2.4* and *2.10*-KO mutants exhibited reduced primary root length and lateral root density in saline conditions, respectively (McLoughlin et al., 2012). SnRK2.4** was shown be involved in regulating the ROS levels in roots in response to cadmium, but remarkably *snrk2.4*-KO mutants displayed an increase in primary root length when exposed to cadmium (Kulik et al., 2012). Overexpression of the SnRK2.4 wheat ortholog (TaSnRK2.4) in *Arabidopsis* caused an increase in primary root growth and resulted in more drought tolerant plants. This was explained by stronger water retention ability in these plants compared to wild-type (Mao et al., 2010). Overexpression of SAPK4 (a rice class 1 SnRK2 ortholog) increased tolerance to salinity and oxidative stress (Diedhiou et al., 2008).

## INTERACTORS OF CLASS 1 SnRK2 KINASES AND THE ROLE OF PA HEREIN

*Arabidopsis* SnRK2.4 is rapidly and transiently activated in saline conditions and is targeted to punctate structures in epidermal and cortex cells in roots (McLoughlin et al., 2012). Activity of class 1 SnRK2 kinases is not directly regulated by PA (Testerink et al., 2007), therefore PA might spatially facilitate protein–protein interactions. Several proteins that interact or are regulated by class 1 SnRK2 kinases also bind to PA (**Figure 1B**).

SnRK2 class 3 kinases are activated by ABA through suppression of protein phosphatases of the PP2C family, including the known PA target ABI1 (Zhang et al., 2004; Yoshida et al., 2006; Umezawa et al., 2009; Vlad et al., 2009; Soon et al., 2012). Although class 1 SnRK2s also have auto-activating capacity, their phosphorylation mechanism has been shown to be different (Burza et al., 2006; Vlad et al., 2010), and it remains to be established if they are inactivated through PP2C phosphatases or other protein phosphatases (Kulik et al., 2011). Several minutes after their activation by salt, class 1 SnRK2 kinases are inactivated again in *Arabidopsis* roots (McLoughlin et al., 2012), possibly through an interaction with the SnRK2-interacting Calcium Sensor (SCS) which inhibits SnRK2 kinase activity (Bucholc et al., 2011), but no evidence for a role of PA herein has been reported.

Nitric oxide (NO) activates a SnRK2.4 ortholog in tobacco; NtOSAK, but no *S*-nitrosylation of NtOSAK was observed after NO treatment. GAPDH was identified as a molecular partner of NtOSAK and *S*-nitrosylation of GAPDH in a NO dependent manner occurred several minutes after applying NO. Although *S*-nitrosylation of GAPDH did not influence the activity of NtOSAK it is speculated that it might be important for the recognition of cellular partners or substrates (Wawer et al., 2010). In addition to SnRK2, oxidized GAPDH binds to PLDδ, which increases the activity of PLDδ, effectively inducing PA formation and forming a feedback loop (Guo et al., 2012).

So far, no *in vivo* phosphorylation targets have been identified for the class 1 SnRK2 members yet, but a preferential phosphorylation affinity peptide motif was identified for SnRK2.10, which is conserved in dehydrins (Vlad et al., 2008). Dehydrins are important for cold, salt, and drought stress and function in protecting macromolecules besides several other potential protective roles (Allagulova Ch et al., 2003) and have been implicated in maintaining root growth in saline conditions (Ruibal et al., 2012). Interestingly, they have affinity for different phospholipids, including PA (Koag et al., 2003; Koag et al., 2009; Eriksson et al., 2011). Although the phosphorylation event still has to be confirmed *in vivo*, this is a candidate class 1 SnRK2 target to control maintenance of root growth in saline conditions.

Summarizing, many of the proteins that interact with or are targeted by class1 SnRK2s have also been described to have PA binding affinity. While PA does not directly influence the activity of class 1 SnRK2 kinases, recruitment of SnRK2.4 to the membrane is speculated be necessary to facilitate interaction with its cellular partners or to phosphorylate target proteins that function near or on the membrane (**Figure 1B**). Alternatively, PA-binding could play an indirect role in the rapid inactivation of the SnRK2 protein kinases through compartmentalization, which was found to occur within minutes after activation by salinity in *Arabidopsis* roots (McLoughlin et al., 2012).

## CONCLUSION AND OUTLOOK

In conclusion, PA is a central phospholipid intermediate which is rapidly and transiently formed during salt and osmotic stress. PA accumulation is used as an appropriate and general sensor to monitor the extracellular environment, which could explain the versatile role of this phospholipid. The specificity in which phospholipases transduce diverse signals remains largely unknown, although differences can be expected through abundance, localization, and substrate preference of the different phospholipases. Besides evidence that PA acts as a docking site for protein recruitment to the membrane, an increasing amount of papers indicate post-translational modifications, activation/inactivation or proteolytic cleavage upon binding either directly (solely in the presence of PA) or indirectly (orchestrated with different proteins), profoundly increasing the complexity of its role. The localization of the PLDs and the effect of PLD-KO and OE lines on the functionality and localization of PA-targets have to be studied to verify their exact relation, which will ultimately result in the understanding of how PA-signaling mediates root proliferation in adverse conditions.

## Conflict of Interest Statement

The authors declare that the research was conducted in the absence of any commercial or financial relationships that could be construed as a potential conflict of interest.
